# The RIPI-f (Reporting Integrity of Psychological Interventions delivered face-to-face) checklist was developed to guide reporting of treatment integrity in face-to-face psychological interventions

**DOI:** 10.1016/j.jclinepi.2022.07.013

**Published:** 2022-08-01

**Authors:** Jesus Lopez-Alcalde, Ninib Yakoub, Markus Wolf, Thomas Munder, Erik von Elm, Christoph Flückiger, Christiane Steinert, Sarah Liebherz, Jenny Rosendahl, Claudia M. Witt, Jürgen Barth

**Affiliations:** a Institute for Complementary and Integrative Medicine, University Hospital Zurich and University of Zurich, Zurich, Switzerland; b Faculty of Health Sciences, Universidad Francisco de Vitoria (UFV), Madrid, Spain; c Instituto Ramón y Cajal de Investigación Sanitaria (IRYCIS), Unidad de bioestadística clínica, Hospital Universitario Ramón y Cajal, (CIBERESP), Madrid, Spain; d Department of Psychology, University of Zurich, Zurich, Switzerland; e Cochrane Switzerland, Centre for Primary Care and Public Health (Unisanté), University of Lausanne, Lausanne, Switzerland; f International Psychoanalytic University Berlin (IPU), Berlin, Germany; g Department of Psychotherapy and Psychosomatics, Justus Liebig University Giessen, Giessen, Germany; h Department of Psychotherapy, University Medical Center Hamburg-Eppendorf, Hamburg, Germany; i Institute of Psychosocial Medicine, Psychotherapy and Psychooncology, Jena University Hospital, Jena, Germany; j Universitätsmedizin Berlin, Corporate Member of Freie Universität Berlin, Humboldt-Universität zu Berlin, and Berlin Institute of Health, Institute of Social Medicine, Epidemiology and Health Economics, Berlin, Germany; k Center for Integrative Medicine, University of Maryland School of Medicine, Baltimore, MD, USA

**Keywords:** Face-to-face psychological intervention, Non-pharmacological intervention, Fidelity, Adherence, Delphi survey, Reporting guideline

## Abstract

**Objectives:**

Intervention integrity is the degree to which the study intervention is delivered as intended. This article presents the RIPI-f checklist (Reporting Integrity of Psychological Interventions delivered face-to-face) and summarizes its development methods. RIPI-f proposes guidance for reporting intervention integrity in evaluative studies of face-to-face psychological interventions.

**Study Design and Setting:**

We followed established procedures for developing reporting guidelines. We examined 56 documents (reporting guidelines, bias tools, and methodological guidance) for relevant aspects of face-to-face psychological intervention integrity. Eighty four items were identified and grouped as per the template for intervention description and replication (TIDieR) domains. Twenty nine experts from psychology and medicine and other scholars rated the relevance of each item in a single-round Delphi survey. A multidisciplinary panel of 11 experts discussed the survey results in three online consensus meetings and drafted the final version of the checklist.

**Results:**

We propose RIPI-f, a checklist with 50 items. Our checklist enhances TIDieR with important extensions, such as therapeutic alliance, provider’s allegiance, and the adherence of providers and participants.

**Conclusion:**

RIPI-f can improve the reporting of face-to-face psychological interventions. The tool can help authors, researchers, systematic reviewers, and guideline developers. We suggest using RIPI-f alongside other reporting guidelines.

## 1. Introduction

Intervention integrity (hereafter “integrity”) is the degree to which the study intervention is delivered as intended [1–4]. It comprises aspects such as what intervention was delivered, how, and to which study participants. The terminology varies among disciplines, including integrity, fidelity, and adherence [5]. Observe the glossary in Appendix 1.

Systematic and transparent reporting of integrity is crucial for several reasons. First, intervention integrity is necessary for the internal validity of studies determining the efficacy of psychological interventions. Compromised integrity can bias the study results, which hampers knowing if the observed effects can be causally attributed to the applied interventions [1,6–9]. Second, varying integrity levels may reduce statistical power and, thus, lead to nonsignificant results [10,11]. Third, integrity informs the external study validity, that is, the degree to which the findings generalize to the real world. Therefore, readers must know the intervention’s integrity to judge whether the study findings apply to their practice [12,13]. In this line, it is essential to know if a psychological intervention is effective only when delivered with high levels of treatment integrity [14]. Fourth, knowing the integrity of the psychological interventions delivered in a trial, both in the intervention and comparator groups, is needed to judge the fairness of treatment comparisons. Understanding the comparison group explains the observed efficacy; minimal care in the control group associates greater effect sizes [15]. However, there is much room for improvement in the reporting of treatment as usual (TAU), the most frequently used control group in psychotherapy trials for depression [16]. Fifth, information about integrity is essential in evidence synthesis, particularly to assess performance bias (bias due to deviations from intended interventions) and the external validity of the evidence [17].

Psychological interventions are interpersonal or informational activities that target biological, behavioral, cognitive, emotional, interpersonal, social, or environmental factors to improve health and wellbeing [18]. Examples are health education, lifestyle interventions, and psycho-therapy. Face-to-face psychological interventions are delivered in the same physical space where the provider and the participant interact [19].

Assessing integrity in face-to-face psychological interventions is more complex than it is in many medical interventions. First, face-to-face psychological interventions are collaborative [1,20], involving therapist-patient interactions at different levels, including single, couple, family, and group settings. Second, integrity depends on several factors, such as the providers’ and recipients’ behaviors, skills, and experiences [13,21]. Notably, patients’ motivation and therapists’ allegiance to treatment are assumed as necessary conditions for effective treatments [22,23]. Third, psychological interventions use theories different from those underpinning medical interventions and established medical concepts (like dose-response relationship) do not always apply [13,24]. Fourth, while a framework for assessing intervention delivery in behavioral intervention trials is available [7], there is neither an equivalent for psychological interventions nor corresponding guidance for reporting.

Although integrity is central to evaluating, comparing, and implementing psychological interventions [11] and researchers in psychology recognize its relevance, it is rarely verified and reported [4,10,12,25,26]. Integrity is described in just 6% of psychological intervention articles [10], so its reporting requires urgent improvement. Several factors can explain this poor reporting. First, integrity definitions in psychology research fail to include all relevant components [7,10]. Second, there are no precise editorial requirements [4] and integrity reporting guidelines specific to psychological interventions are lacking. Although CONSORT-SPI 2018 and TIDieR [12,13] provide general helpful advice, in our opinion, they do not cover all the key actors and components relevant for the integrity of psychological interventions, for example, nonspecific intervention aspects like therapeutic alliance or expectations.

The RIPI-f (Reporting Integrity of Psychological Interventions delivered face-to-face) checklist was developed by consensus. RIPI-f will be a helpful tool for health researchers writing manuscripts of studies evaluating the effects of face-to-face psychological interventions. It proposes a clear list of items that should be reported to ensure the readers know the integrity of face-to-face psychological interventions. This article aims to (1) describe the methods used to develop the checklist and (2) present the checklist.

## 2. Development of the checklist

**Fig. 1.**
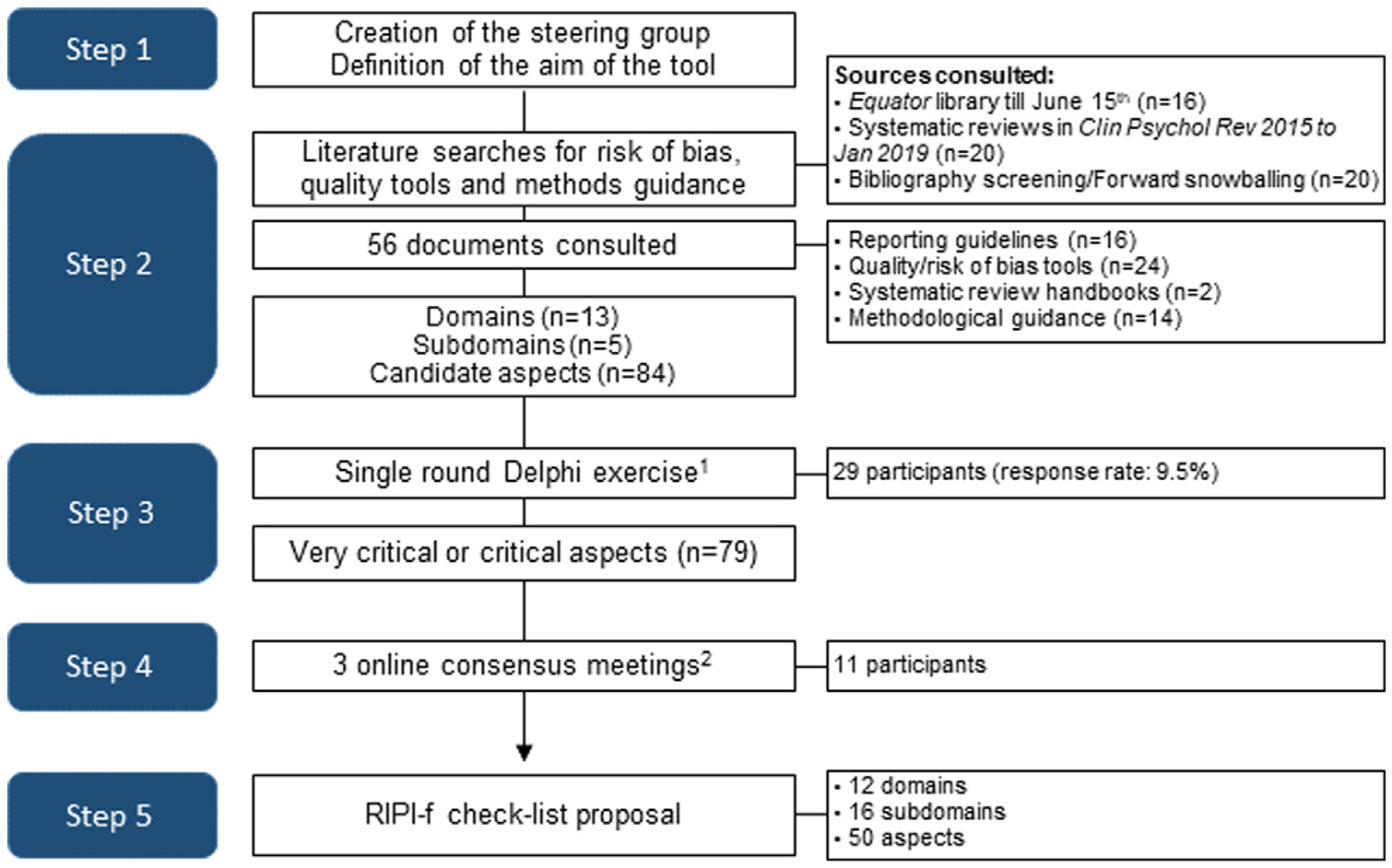
Stepwise procedure to develop the RIPI-f checklist. ^1^Delphi exercise: July 20 to August 13, 2020; ^2^Online consensus meetings: September 23, October 13, and December 8, 2020 (4 h each).

We implemented a five-step procedure (Fig. 1) following established methods [27]. First, the steering group (S.G.) (J.B., N.Y., and J.L.A.) defined the checklist purpose. Second, we consulted the following sources for risk of bias/quality tools and guidance for measuring and reporting integrity: (a) EQUATOR until June 2020 (psychology, psychiatry, public health, and behavioral medicine); (b) quality tools used in systematic reviews published in Clin Psychol Rev 2015 to January 2019; and (c) bibliographies of relevant articles and forward snowballing. The SG assessed 56 documents (Appendix 2), identified 84 candidate aspects potentially relevant for intervention integrity, and organized them in 13 domains and five subdomains (Appendix 3) as per the TIDieR structure [12]. Third, a multidisciplinary group of 29 experts (psychologists with different psychotherapy orientations and physicians, all of them with research experience) completed a single-round online Delphi survey (experts invited: 304; response rate: 9.5%). They rated the relevance of each item on a 9-point Likert scale as very important (7–9 points), important (4–6 points), or not important (1–3 points). The respondents indicated their confidence in each rating (high, moderate, or low) and could comment or propose new items. The SG analyzed the survey results and classified the relevance of each item as very critical, critical, not critical, or unclear (Box 1). Fifty six percent (47/84) of the items were defined as ‘very critical’, 38% (32/84) as ‘critical’, and 6% (5/84) as unclear. Because no item was deemed ‘not critical’, there was only one Delphi round.

The S.G. held three online consensus meetings. The 11 attendees (nine also participated in the survey) were psychologists and physicians, all of them with research experience. They discussed the survey procedures and results, such as the decision to conduct only one Delphi round. They also commented, organized, and added items to the initial list and approved its final version. Decisions were adopted by consensus and voting (two-thirds of the votes needed for approval). The manuscript draft was circulated via email among the meeting participants. The S.G. incorporated their feedback and approved the final version of the manuscript.

## 3. The RIPI-f checklist

### 3.1. Scope

RIPI-f aims to improve the reporting of intervention integrity in evaluative studies of face-to-face psychological interventions. Our tool focuses on the face-to-face setting and excludes digital interventions, such as cognitive-behavioural therapy via smartphone. In face-to-face psychological interventions, the interaction between provider and participant is vital. Also, digital interventions may merit an extension of the checklist [28,29].

The checklist includes critical items that authors should report separately for each study arm with a face-to-face psychological intervention, including control groups, such as those defined as TAU. Thus, although our checklist does not consider the assessment of the fairness of treatment comparisons, a straightforward reporting of the intervention integrity in both study arms is a required step toward this aim.

RIPI-f applies to protocols of evaluative studies or their full reports, such as randomized controlled trials, nonrandomized trials, or observational studies. RIPI-f complements relevant reporting guidelines, particularly TIDieR [12], CONSORT-SPI 2018 Extension [13], SPIRIT 2013 [30], and TREND [31].

### 3.2. Contents

Table 1 presents RIPI-f. The checklist includes 50 items, divided into 12 domains and 16 subdomains. We tried to maintain the TIDieR domains but elaborated or added domains, subdomains, and items where necessary.

### 3.3. Use of the RIPI-f checklist

RIPI-f proposes critical aspects that authors should report to describe the integrity of face-to-face psychological interventions, whether they are part of the experimental condition or the control condition. A thorough description of the comparator will help explain the magnitude of the observed effects [12]. We suggest that authors consider the checklist at the study planning stage.

Users of RIPI-f for a given study report or protocol may judge whether each checklist item is “reported”, “not reported”, or “not applicable”. In addition, the checklist addresses the intervention’s delivery plan and the observed delivery, which helps in judging whether the intervention differed from the plan—a critical component of intervention integrity [1–4]. It may not be possible to report all the information in the printed report. In that case, authors may present an expanded description in locations beyond the primary article, such as supplementary material with a stable location [12,13,32,33]. Journals and publishers may endorse the use of RIPI-f and refer to the checklist in the “Instructions to authors”.

As RIPI-f applies to any evaluative study, we suggest using it with the relevant reporting guideline, particularly CONSORT-SPI, SPIRIT, TREND, and STROBE [13,30, 31,34]. When authors consider aspects corresponding to interventions, for example, item 5 of the CONSORT-SPI checklist, they could refer to RIPI-f. RIPI-f enhances TIDieR [12]; there is no need to use both. Box 2 and Appendix 4 describe the main differences from TIDieR. Appendix 5 presents terminology applied to a behavior change intervention trial.

## 4. Discussion

### 4.1. Summary

We developed guidance for reporting intervention integrity in evaluative studies of face-to-face psychological interventions. RIPI-f covers critical aspects concerning integrity: the intervention providers, the participants receiving the intervention, how and where the intervention was delivered, when and how often the intervention was delivered, whether the intervention was tailored or modified during the study, methods to assess adherence, and the actual adherence. RIPI-f enhances TIDieR and can be used in conjunction with other reporting guidelines, such as CONSORT-SPI [13], STROBE [34], SPIRIT [30], and TREND [31].

### 4.2. Potential beneficiaries of the checklist

The use of RIPI-f can help several stakeholders. First, authors can submit manuscripts with a better description of integrity. Second, journal editors and peer-reviewers will probably receive better submissions after completion of the checklist. Third, RIPI-f can help researchers improve their study designs and adequately plan intervention integrity. Fourth, knowing integrity can help readers interpret the study results, such as explaining whether the absence of effect could be due to low integrity or whether unplanned interventions contributed to effectiveness [6,41]. Fifth, researchers can consider the observed integrity to design future interventions to meet participants’ needs [8,11].

The use of RIPI-f can help systematic reviewers extract information relevant in several ways. The first is to assess performance bias when using a risk of bias tool; RIPI-f can help reviewers verify whether the participants received the planned intervention within each study arm, which is critical to judge if the comparison was fair [7]. The second is to interpret heterogeneity in the study results [35] and assess their external validity, which is essential to grading the certainty of the evidence [17].

Adequate integrity reporting will also benefit the article readers (providers, consumers, policymakers, guideline developers, and other decision-makers) in terms of assessing the trustworthiness and replicability of the intervention in their setting. Integrity relates to the feasibility and acceptability of interventions [8,17], information needed to make meaningful comparisons of the available interventions, and informed decisions [11]. Thus, it can also help guideline developers formulate appropriate recommendations for practice [42,43] and, consequently, facilitate the implementation of research findings in clinical practice [6]. Finally, better reporting of psychological intervention integrity may alleviate the crisis of replicability of findings in clinical psychology research [44,45]. For example, variation in TAU intensity can impact the outcome of trials and bias estimates of psychotherapy efficacy [16,46]; RIPI-f can also help improve the reporting of these comparators and therefore help study their impact on the intervention effects.

### 4.3. Strengths and limitations

We developed RIPI-f using rigorous methods [27] and our exhaustive searches likely captured all the relevant components of psychological intervention integrity.

The Delphi response rate (9.5%) was lower than similar reporting guidelines, such as TIDieR-Placebo (31%) [47]. Consequently, the checklist may have been influenced by the authors’ perspectives (who were also consensus meetings experts). The specificity of our topic and the long list of items in the survey could explain this low participation. We invited colleagues as per their general background in clinical psychology research and systematic reviews but not as per their potential interest in intervention integrity. Consequently, some experts may have declined due to their lack of interest/competence in the topic. We did not invite a broader community, such as full-time practitioners, consumers, or experts from similar intervention fields, like educational science or social work. We preferred to set up a group specialized in research methods and various theoretical orientations in clinical psychology to capture their views concerning methodological considerations.

The main limitation of the RIPI-f checklist is its extension, which may reduce feasibility. As with other reporting guidelines [13], the survey participants defined most items as potentially relevant (*n* = 79; 94%). Several factors may explain this high proportion of relevant items. The survey took many items from risk of bias tools and published reporting guidelines, so other researchers had already considered them relevant. Moreover, there is no well-established definition of the integrity of psychological interventions [7]; participants may have refrained from excluding items due to this concept ambiguity. On the other hand, the RIPI-f checklist may be criticized as looking at psychological interventions as drugs, thus disregarding their dynamic and interactional nature. While the researcher’s allegiance toward a specific intervention may be seen as a performance bias (i.e., exaggeration of effects because the provider knows the intervention being delivered), the therapist’s allegiance is a vital component of delivering a specific intervention. Similarly, bias from a deviation from the intended intervention is likely to happen if therapists have no allegiance to the treatment.

It can be argued that the therapeutic alliance, or expectations, is not part of treatment integrity per se but rather intermediate outcomes on the process level. The inclusion of process-like aspects was a topic of discussion during our consensus meetings. We decided to include these constructs because the breakdown of any of these aspects can seriously compromise treatment integrity. Besides, we aimed to compile all relevant items in one checklist; therefore, following other authors’ approaches and previous frameworks [3,6,7,10], we incorporated these constructs into the RIPI-f checklist.

### 4.4. Implications for practice and research

We will make RIPI-f available in Open Science Framework and submit the checklist for inclusion in the EQUATOR Library and goodreports.org to enhance dissemination. We will also disseminate the tool through our institutions’ channels.

The RIPI-f checklist is a work in progress and we encourage feedback and further discussion, particularly proposals to reduce its extension. We will test the tool for feasibility and invite stakeholders to provide feedback. Once the next version of the checklist is available, we will elaborate a report explaining each checklist item with examples of transparent reporting. We will welcome translations and empirical studies evaluating the impact of the final tool.

Adhering to RIPI-f might be time-consuming and increase the manuscript length. However, we consider that not investing effort in reporting intervention integrity is more costly than doing so [6]. Better reporting is vital to addressing integrity’s complexity, increasing internal and external study validity, and allowing replicability, a critical challenge in research on psychological interventions [44]. On the other hand, the manuscript can still be concise through the efficient use of the tool and details on the intervention provided as supplementary material.

We acknowledge an urgent need to define valid measurements for several checklist items. Examples are the therapist’s allegiance or expectations. In this line, a framework for quantitatively assessing the integrity of psychological interventions is also needed. For example, a score per study arm summarizing intervention integrity would help identify fair comparisons. The task is challenging, as the model variables, and their relative weight are not agreed upon [7,23]. In addition, the importance of each item can vary depending on the study, intervention, and outcome [35]. For example, supervision may be less critical in simple interventions or pragmatic trials.

Finally, further research is required to demonstrate whether the completion of RIPI-f by authors improves reporting, for example, if the tool reduces the risk of bias domains flagged as unclear in systematic reviews due to poor reporting.

## 5. Conclusion

The RIPI-f checklist proposes guidance for reporting intervention integrity in evaluative studies of face-to-face psychological interventions. It enhances TIDieR by addressing psychological interventions specifically. The checklist can help trialists plan their studies, derive valid conclusions, and facilitate the transfer of effective interventions into clinical practice. RIPI-f is a work in progress that may require feedback and revision in the future.

## Supplementary Material

1

2

3

4

5

## Figures and Tables

**Table 1.** The RIPI-f checklist

Domain	Item	Item brief name and explanation	Planned^a^	Observed^b^
**1. Intervention brief name**				
	1	**Intervention brief name:** A name or phrase that describes the intervention.	NA	
**2. Why**				
	2	**Rationale:** Justification of why the intervention can work (any rationale, theory, or goal of the components essential to the intervention/s).	NA	
**3. What**				
	3	**Main intervention/s:** (a) Main intervention/s and components; (b) Manual (or protocol) for delivery (with reference, online appendix, or URL, preferably with a permanent link).		
	4	**Materials:** Physical or informational materials used by providers and participants (with reference, online appendix, or URL, preferably with a permanent link).		
	5	**Co-interventions:** Provision of additional care (content, materials, and procedures).		
**4. Who: Intervention provider** (person/s delivering the intervention/s)				
**Nr. Providers**				
	6	**Nr. providers:** (a) Total nr. providers in the study; (b) Nr. participants per provider; (c) Nr. providers per participant; (d) Delivery in individual or group sessions (if applicable, nr. participants and providers per group).		
**Professional competencies:** A competency is a knowledge, skill, or attitude shown by the provider that enables to effectively perform an activity to the expected standard.				
	7	**Professional competencies at study entry:** (a) General qualification^c^; (b) Experience with the study intervention, such as years or nr. participants treated.		
	8	**Training for the intervention:** (a) Training of providers^d,e^; (b) Assessment of providers’ competencies post-training^f,g^; (c) Acceptable competencies threshold.		
	9	**Providers’ supervision during the study:** (a) Supervision during the study^h^; (b) Acceptable performance threshold.		
	10	**Strategies for handling providers below standards** ^i^		
**Providers’ allegiance to the intervention:** Provider’s professional preference for the intervention model, which deems it superior to other models of intervention.				
	11	**Providers’ allegiance to the intervention** ^f,g^		
**Providers’ motivation:** Extent to which a provider is inclined to work with a particular participant.				
	12	**Providers’ motivation** ^f,g^		
**Therapeutic alliance:** A cooperative working relationship between the provider and participant. It consists of 3 components: agreement on the treatment goals, agreement on the tasks, and development of a personal bond.				
	13	Therapeutic alliance^f,g^		
**Providers’ awareness of being observed:** If the providers know they are observed, they may change their behavior.				
	14	**Providers’ awareness of being observed:** (a) Providers’ knowledge of being observed; (b) Observation methods, if applicable (recording, direct observation, etc.).		
**5. Who: Participant** (person receiving the intervention)				
**Participants’ receipt of the intervention:** Degree to which the participants understand and can use the intervention skills during the study.				
	15	**Participants’ comprehension of the intervention** ^f,g^		
	16	**Strategies to increase participants’ comprehension**		
**Participants’ enactment of the intervention:** Extent to which the participants can use the intervention skills in a relevant real-life setting.				
	17	**Participants’ enactment** ^f,g^		
	18	**Strategies to increase participants’ enactment**		
**Participants’ expectations:** Cognitions about treatment-related health outcomes in the future after a specific intervention.				
	19	**Participants’ expectations** ^f,g^		
	20	**Strategies to increase participants’ expectations**		
**Participants’ awareness of being observed:** If the participants know they are being observed, they may change their behavior.				
	21	**Participants’ awareness**^f,g^: (a) Participants’ knowledge of being observed; (b) Detail observation methods, if applicable (recording, observation, etc.).		
**Participants’ monitoring during the study** (assessment and handling of noncompliant participants)				
	22	**Participants’ monitoring during the study:** (a) Monitoring during the study^h^; (b) Acceptable performance threshold.		
**Strategies for handling participants below standards**				
	23	**Strategies for handling noncompliant participants’**		
**6. How:** How the main intervention is delivered				
	24	**How:** (a) Procedures: Activities or processes for the intervention; (b) Enabling or supporting activities; (c) Mode of delivery: State that the intervention is delivered face-to-face.		
**7. Where:** The location description can help consider the site effects (interventions may be implemented differently across sites).				
	25	**Locations of the interventions:** (a) Nr. sites involved in the study (monocentric/multicentric, if multicentric, detail the nr. sites); (b) Nr. sites that each participant had to attend; (c) Types of sites. Examples: Outpatient or inpatient setting or the participant’s home; (d) Necessary infrastructure or relevant features.		
**8. When and how much**				
	26	**When – Intervention timing:** (a) Intervention period (such as from March to August); (b) Scheduling of sessions (time between sessions); (c) Total intervention period (days or months). If possible, present a graphical presentation depicting the flow and timing of the sessions.		
	27	**How much – Dose of the intervention:** (a) Length of each session (minutes); (b) Total nr. sessions; (c) Minimal nr. sessions to attend (if applicable).		
**9. Tailoring of the intervention:** The intervention includes elements adapted to the individual needs of each participant. Tailoring occurs at the participant level, so not all the participants receive an identical intervention. The provider can adhere to the manual but still incorporate flexibility in therapeutic technique and style by adjusting certain features as per the participant’s individual needs.				
	28	**Tailoring characteristics** ^j^		
**10. Legitimate intervention modifications:** Allowed changes at the study level (not individual tailoring).				
	29	**Legitimate modifications**^j^: For example, report if the trial allowed substantial variation across sites in multicentric studies.		
**11. How well** (planned): The plan to assess the integrity and how it was finally assessed.				
	30	**Critical items for intervention integrity** (as defined by the study authors)		NA
	31	**Assessment of the providers’ adherence**^k^: Degree to which the providers deliver the planned intervention procedures (and avoid proscribed procedures).		
	32	**Assessment of the intervention differentiation**^k^: Extent to which the interventions under investigation differ from each other over critical dimensions in the intended manner.		
	33	**Assessment of the participants’ adherence**^k^: Degree to which the participants perform the planned intervention (and avoid proscribed procedures) as planned. Any intervention change agreed upon with care providers or investigators but not permitted by the trial protocol is also considered a deviation.		
**12. How well** (actual)				
**Actual adherence of providers**				
	34	**Deviations in professional competencies:** (a) Deviations in training (more/less intense than planned); (b) Deviations in supervision (more/less intense than planned).	NA	
	35	**Errors of commission or omission:** (a) Errors of commission: Adding interventions (or cointerventions) not specified by the protocol; (b) Errors of omission: Deleting interventions (or cointerventions) that were specified by the protocol.	NA	
	36	**Deviations in the numbers of providers:** (a) Deviation in the nr. providers by participant (such as lower nr. providers by participant); (b) Providers who decided to discontinue the intervention; (c) Delivery to a group of participants (instead of individually) or vice versa.	NA	
	37	**Deviations in the mode of delivery:** Internet-based instead of face-to-face.	NA	
	38	**Deviations in the intervention location:** For example, if the intervention was planned to be delivered at the hospital but was ultimately delivered at home.	NA	
	39	**Deviations in the intervention timing:** (a) Intervention period; (b) Scheduling of contact sessions (time between sessions); (c) Total duration of the intervention period (days or months).	NA	
	40	**Deviations in the intervention dose:** (a) Length of each session (minutes); (b) Total nr. Sessions.	NA	
	41	**Deviations in the planned tailoring and accepted modifications during the study**	NA	
**Actual intervention differentiation**				
	42	**Actual intervention differentiation**	NA	
	43	**Contamination across treatment/control conditions** ^l^	NA	
**Actual adherence of study participants**				
	44	**Deviation in the preparation for the intervention**^m^: More/less intense than planned.	NA	
	45	**Errors of commission or omission:** (a) Errors of commission: Adding interventions, cointerventions, or behavior not specified by the protocol; (b) Errors of omission: Deleting interventions (or cointerventions) that were specified by the protocol.	NA	
	46	**Deviations in the numbers of participants:** Nr. participants’ that decided to discontinue the intervention.		
**Overall summary of the actual intervention integrity** (as per the critical items defined in item 30)				
	47	**Overall % of providers with compromised intervention integrity within each study arm**	NA	
	48	**Overall % of participants with compromised intervention integrity within each study arm**	NA	
	49	**Verification of intervention differentiation** (whether the treatment conditions differed in the intended manner)	NA	
	50	**Overall judgment on the intervention integrity**	NA	

NA, not applicable; NR, not reported; Nr, number; R, reported; %, percentage.

a Possible answers: R, NR or NA. Also, detail the location in the text.

b Possible answers: R, NR or NA. Also, detail the location in the text. Detail if there were relevant differences between the observed situation and the plan.

c (a) Academic background, such as degree, master, or PhD; (b) Profession, such as psychologist, nurse, etc.; (c) General professional experience, such as years or nr. participants attended; (d) % of providers in training.

d (a) Presence (yes/no); (b) Content; (c) Trainer: internal or external, experience, etc. (d) Modality: indirect training (didactic instructions and written materials) or direct training (opportunities for practice, such as role-playing); (e) When: punctually or continuous during the trial; (f) Intensity: nr. and duration of sessions; (g) Materials; and (h) Standardization of training across providers.

e Observed aspects: (a) Overall % of providers fully trained; (b) Differences in levels of training among providers, centers (in multicentric studies), and over time.

f (a) Presence of assessment (yes/no); (b) Content: what is assessed; (c) How the assessment is done, such as the measurement tool; (d) Who measures: self-assessment, internal or external observer and blinding status to the allocated intervention; and (e) When: punctually or continuously during the trial.

g Observed aspects: (a) Overall level per study arm; (b) Differences in levels among providers, centers (in multicentric studies), and over time.

h (a) Presence of supervision or monitoring (yes/no); (b) Content: what is considered; (c) How the assessment is done, such as the measurement tool; (d) Who assesses: self-assessment, internal or external observer and blinding status to the allocated intervention; and (e) When: punctually or continuously during the trial.

i (a) Presence of correction procedures (yes/no). For example, strategies for providers dropping out, such as having a pool of trained providers ready to join, or strategies for participants missing a session, such as instruction on how to use the booklet session and practice that session content; (b) Observed overall % of corrected providers or participants; (c) Observed differences in the % of corrected providers or participants among centers (in multicentric studies) and over time.

j (a) Presence (yes/no); (b) Why: justify the need of tailoring/modification; (c) What (content): elements tailored/modified; (d) How; (e) When.

k (a) Presence (yes/no); (b) Content: what elements are assessed; (c) Who assesses: self-assessment, internal or external observer, observer’s experience and blinding status to the allocated intervention; (d) How the assessment is done: direct observation or video or audio recording, indirect assessments, such as providers’ self-reports, interviews with providers or participants, completed homework; (e) When the assessment is done: punctually or continuous during the trial.

l Participants in one group receive the treatment or are exposed to the intervention meant solely for the other group, thereby minimizing any real difference between the groups.

m For example, the study protocol required that the patients attended an informative session previously to the intervention start. However, some patients did not attend that session.

## What is new?

### Key findings

- Intervention integrity is the degree to which a study intervention is delivered as intended.
- CONSORT-SPI and TIDieR guidelines already address psychological intervention reporting, but more guidance in describing integrity is needed.

### What this adds to what was known?

- The RIPI-f checklist (see Table 1) proposes the first critical set of items to be reported to allow for an evaluation of intervention integrity in face-to-face psychological interventions.
- The checklist considers the peculiarities of psychological interventions. Examples are the providers’ allegiance to the intervention, motivation, therapeutic alliance, and the participants’ receipt and enactment of the intervention and their expectations.
- The checklist applies to any evaluative study, such as randomized or nonrandomized trials or observational studies.

### What is the implication, what should change now?

- RIPI-f integrates TIDieR and complements other relevant reporting guidelines, such as CONSORT-SPI, SPIRIT, and TREND.
- Adherence to RIPI-f may substantially enhance the reporting of studies evaluating face-to-face psychological interventions.
- RIPI-f should be considered a living document. We encourage piloting, feedback, and further discussion.

## Box 1 Item relevance for the integrity of face-to-face psychological interventions.

Very critical item: At least 50% of respondents had high confidence in defining the item as very important.

Critical item: At least 80% of respondents had moderate or high confidence in defining the item as important or very important.

Not critical item: At least 80% of respondents defined the item as being not important (independent of the confidence in the rating).

Unclear: The survey results did not allow for classifying the item’s relevance.

## Box 2 Main changes of RIPI-f to TIDieR.

1. RIPI-f is more comprehensive and specific than TIDieR: It allows for fine-grained reporting and unambiguous description of face-to-face psychological intervention integrity.
2. Integration of relevant methodological guidance on psychological intervention integrity [6–8,10,11,25,35–40].
3. Differentiation between participants’ and providers’ contributions to integrity.
4. Distinction between planned and observed intervention delivery.
5. Consideration of peculiarities of psychological interventions, such as the provider’s allegiance to the intervention, motivation, and the therapeutic alliance and the participants’ receipt, enactment, and expectations.
6. More detailed description of the intervention provider.
7. Differentiation between the integrity assessment plan and how integrity was finally assessed.
8. Clear guidance to describe the integrity of the cointerventions.
