# Supplementary material for: The RIPI-f (Reporting Integrity of Psychological Interventions delivered face-to-face) checklist was developed to guide reporting of treatment integrity in face-to-face psychological interventions
Source: J Clin Epidemiol. Author manuscript; Available in PMC 2024 Jun 21. (PMC11192047; doi:10.1016/j.jclinepi.2022.07.013)
Supplement: 1 [file NIHMS2000500-supplement-1.docx]

# Appendix 1. Glossary of terms

| **Adherence of participants:** the degree to which the participants perform the allocated intervention (and avoid proscribed procedures) as planned.  **Adherence of providers:** the degree to which the providers follow the planned intervention procedures (and avoid proscribed procedures) during the actual delivery of the intervention [1, 2].  **Allegiance to the intervention:** provider’s professional preference for the intervention model, which deems it superior to other models of intervention. Allegiance influences the provider’s preferences for one intervention studied over another. Allegiant providers may achieve better intervention effects[3].  **Attrition bias:** systematic error in the effect estimate due to systematic differences in the way participants are lost from a study [4].  **Competence:** the habitual and judicious use of knowledge, skills, and attitudes in daily practice for the benefit of the patients. Competence refers to an individual's capacity to perform job responsibilities[5] and is based on the acquisition of a set of competencies (see below) [6, 7].    **Competency:** a knowledge, skill, or attitude that enables one professional to effectively perform an activity to the expected standard[7]. A competency focuses on an individual's actual performance in a particular situation[5].  **Contamination:** the exposure of the participants of one group in a trial to the intervention that is meant solely for the other group. Contamination minimizes any real difference in effect between the groups [8, 9].  **Enactment of intervention:** the extent to which participants can use the learnt intervention skills in relevant real-life settings [10-13].  **Expectations (participant’s expectations):** cognitions about treatment-related health outcomes in the future after a specific intervention [14, 15].  **Face-to-face intervention:** intervention delivered in the same physical space in which both the provider and the participant interact [16].  **Hawthorne effect:** change in the behaviour of subjects participating in a study due to their awareness of being studied [17]. For example, productivity can increase among workers who know they are part of a research study.  **Integrity (intervention integrity, intervention fidelity, implementation fidelity):** the degree to which the intervention of a study is carried out as intended [18-22]. For the sake of brevity, this article uses the term “integrity” alone and defines integrity and fidelity as synonyms. In addition, we consider adherence as just one element of integrity and differentiate between participants’ and providers’ adherence.  **Intervention differentiation:** the extent to which the interventions under investigation differ from each other along critical dimensions in the intended manner [2, 12, 23-25].  **Intervention efficacy:** the extent to which the intervention influences the research endpoint of interest.  **Intervention manual:** specification of the interventions and strategies for its acceptable implementation. The manual describes the intervention process and how to sequence the techniques, provides examples of intervention operations, and indicates procedures for handling deviations [2].  **Missing participant data:** outcome data of a participant in a study that is not available and, therefore, cannot be analyzed for a specific effect estimate [26].  **Non-specific intervention effects:** elements present in all psychotherapeutic interventions, such as a healing setting, education, a treatment rationale, expectations of improvement, a treatment ritual, and the therapeutic relationship [27].  **Performance bias:** systematic error in the effect estimate due to deviations from the intended interventions [28].  **Psychological intervention:** interpersonal or informational activities, techniques, or strategies that target biological, behavioural, cognitive, emotional, interpersonal, social, or environmental factors with the aim of improving health functioning and well-being [29]. This article uses the term intervention, instead of treatment, as the aim of a psychological intervention can be preventing or treating a health problem.  **Receipt of the intervention:** the degree to which the participants understand and can use the intervention skills during the study [10].  **Therapeutic alliance:** a cooperative working relationship between client and therapist, considered by many to be an essential aspect of successful therapy. It consists of three essential elements: agreement on the goals of the treatment, agreement on the tasks, and the development of a personal bond made up of reciprocal positive feelings [30]. An optimal therapeutic alliance is achieved when the patient and therapist share beliefs about the goals of the treatment and view the methods used to achieve these as efficacious and relevant [31]. |
| --- |

**Bibliography**

[1] Waltz J, Addis ME, Koerner K, Jacobson NS. Testing the integrity of a psychotherapy protocol: assessment of adherence and competence. J Consult Clin Psychol. 1993;61:623-30.

[2] Perepletchikova F, Kazdin AE. Treatment integrity and therapeutic change: issues and research recommendations. Clin Psychol Sci Pract. 2005;12:365-83.

[3] Steinert C, Munder T, Rabung S, Hoyer J, Leichsenring F. Psychodynamic therapy: as efficacious as other empirically supported treatments? A meta-analysis testing equivalence of outcomes. Am J Psychiatry. 2017;174:943-53.

[4] Nunan D, Aronson J, Bankhead C. Catalogue of bias: attrition bias. BMJ Evid Based Med. 2018;23:21-2.

[5] McConnell EA. Competence vs. competency. Nursing Management. 2001;32:14.

[6] Epstein RM, Hundert EM. Defining and assessing professional competence. Jama. 2002;287:226-35.

[7] Birnbaum ML, Daily EK. Competency and competence. Prehosp Disaster Med. 2009;24:1-2.

[8] Higgins JPT, Savović J, Page MJ, Elbers RG, Sterne JAC. Chapter 8: Assessing risk of bias in included studies. In: Higgins JPT, Thomas J, Chandler J, Cumpston M, Li T, Page MJ, Welch VA (editors). Cochrane Handbook for Systematic Reviews of Interventions version 6.2 (updated February 2021). Available from <www.training.cochrane.org/handbook>. 2021.

[9] Stone JC, Glass K, Clark J, Ritskes-Hoitinga M, Munn Z, Tugwell P, et al. The MethodologicAl STandards for Epidemiological Research (MASTER) scale demonstrated a unified framework for bias assessment. J Clin Epidemiol. 2021;134:52-64.

[10] Leeuw M, Goossens ME, de Vet HC, Vlaeyen JW. The fidelity of treatment delivery can be assessed in treatment outcome studies: a successful illustration from behavioral medicine. J Clin Epidemiol. 2009;62:81-90.

[11] Lichstein KL, Riedel BW, Grieve R. Fair tests of clinical trials: a treatment implementation model. Adv Behav Res Ther. 1994;16:1-29.

[12] Borrelli B, Sepinwall D, Ernst D, Bellg AJ, Czajkowski S, Breger R, et al. A new tool to assess treatment fidelity and evaluation of treatment fidelity across 10 years of health behavior research. J Consult Clin Psychol. 2005;73:852-60.

[13] Bellg AJ, Borrelli B, Resnick B, Hecht J, Minicucci DS, Ory M, et al. Enhancing treatment fidelity in health behavior change studies: best practices and recommendations from the NIH Behavior Change Consortium. Health Psychol. 2004;23:443-51.

[14] Crow R, Gage H, Hampson S, Hart J, Kimber A, Thomas H. The role of expectancies in the placebo effect and their use in the delivery of health care: a systematic review. Health Technol Assess. 1999;3:1-96.

[15] Barth J, Kern A, Lüthi S, Witt CM. Assessment of patients’ expectations: development and validation of the Expectation for Treatment Scale (ETS). BMJ Open. 2019;9:e026712.

[16] Suh H, Sohn H, Kim T, Lee DG. A review and meta-analysis of perfectionism interventions: Comparing face-to-face with online modalities. J Couns Psychol. 2019;66:473-86.

[17] McCambridge J, Witton J, Elbourne DR. Systematic review of the Hawthorne effect: New concepts are needed to study research participation effects. Journal of Clinical Epidemiology. 2014;67:267-77.

[18] Yeaton WH, Sechrest L. Meaningful measures of effect. Journal of Consulting and Clinical Psychology. 1981;49:766-7.

[19] Perepletchikova F. On the Topic of Treatment Integrity. Clin Psychol (New York). 2011;18:148-53.

[20] Perepletchikova F, Treat TA, Kazdin AE. Treatment integrity in psychotherapy research: analysis of the studies and examination of the associated factors. J Consult Clin Psychol. 2007;75:829-41.

[21] Perepletchikova F, Hilt LM, Chereji E, Kazdin AE. Barriers to implementing treatment integrity procedures: survey of treatment outcome researchers. J Consult Clin Psychol. 2009;77:212-8.

[22] Vermilyea BB, Barlow D, O'Brien GT. The importance of assessing treatment integrity: An example in the anxiety disorders. Journal of behavioral assessment. 1984;6:1-11.

[23] Kazdin AE. Comparative outcome studies of psychotherapy: methodological issues and strategies. J Consult Clin Psychol. 1986;54:95-105.

[24] Moncher FJ PR. Treatment fidelity in outcome studies. Clin Psychol Rev. 1991;11:247e66.

[25] Yeaton WH, Sechrest L. Critical dimensions in the choice and maintenance of successful treatments: strength, integrity, and effectiveness. J Consult Clin Psychol. 1981;49:156-67.

[26] Akl EA, Johnston BC, Alonso-Coello P, Neumann I, Ebrahim S, Briel M, et al. Addressing dichotomous data for participants excluded from trial analysis: a guide for systematic reviewers. PLoS One. 2013;8:e57132.

[27] DeRubeis RJ, Brotman MA, Gibbons CJ. A Conceptual and Methodological Analysis of the Nonspecifics Argument. Clinical Psychology: Science and Practice. 2005;12:174-83.

[28] Sterne JAC, Savovic J, Page MJ, Elbers RG, Blencowe NS, Boutron I, et al. RoB 2: a revised tool for assessing risk of bias in randomised trials. BMJ. 2019;366:l4898.

[29] England MJ, Butler AS, Gonzalez ML, Institute of Medicine (U.S.). Committee on Developing Evidence-Based Standards for Psychosocial Interventions for Mental Disorders, Institute of Medicine (U.S.). Board on Health Sciences Policy. Psychosocial interventions for mental and substance use disorders : a framework for establishing evidence-based standards. Washington, D.C.: The National Academies Press; 2015.

[30] Bordin E. The generalizability of the psychoanalytic concept of the working alliance. Psychotherapy. 1979;16:252-60.

[31] Ardito R, Rabellino D. Therapeutic alliance and outcome of psychotherapy: historical excursus, measurements, and prospects for research. Frontiers in Psychology. 2011;2.
