# Supplementary material for: The RIPI-f (Reporting Integrity of Psychological Interventions delivered face-to-face) checklist was developed to guide reporting of treatment integrity in face-to-face psychological interventions
Source: J Clin Epidemiol. Author manuscript; Available in PMC 2024 Jun 21. (PMC11192047; doi:10.1016/j.jclinepi.2022.07.013)
Supplement: 2 [file NIHMS2000500-supplement-2.docx]

# Appendix 2. Documents consulted to identify the survey aspects related with intervention integrity

We assessed 56 documents for aspects potentially relevant for the integrity of face-to-face psychological interventions.

***a. Reporting guidelines (n=16)***

1. Appelbaum M, Cooper H, Kline RB, et al. Journal article reporting standards for quantitative research in psychology: The APA Publications and Communications Board task force report. Am Psychol. 2018;73:3-25.
2. Borek AJ, Abraham C, Smith JR, et al. A checklist to improve reporting of group-based behaviour-change interventions. BMC Public Health. 2015;15:963.
3. Boutron I, Moher D, Tugwell P, et al. A checklist to evaluate a report of a nonpharmacological trial (CLEAR NPT) was developed using consensus. J Clin Epidemiol. 2005;58:1233-40.
4. Chan AW, Tetzlaff JM, Altman DG, et al. SPIRIT 2013 statement: defining standard protocol items for clinical trials. Ann Intern Med. 2013;158:200-7.
5. Davidson KW, Goldstein M, Kaplan RM, et al. Evidence-based behavioral medicine: what is it and how do we achieve it? Ann Behav Med. 2003;26:161-71.
6. Des Jarlais DC, Lyles C, Crepaz N, *et al*. Improving the reporting quality of nonrandomized evaluations of behavioral and public health interventions: the TREND statement. Am J Public Health. 2004;94:361-6.
7. von Elm E, Altman DG, Egger M, et al. Strengthening the reporting of observational studies in epidemiology (STROBE) statement: guidelines for reporting observational studies. BMJ. 2007;335:806-8.
8. Hoffmann TC, Glasziou PP, Boutron I, et al. Better reporting of interventions: template for intervention description and replication (TIDieR) checklist and guide. BMJ. 2014;348:g1687.
9. Leech NL, Onwuegbuzie AJ. Guidelines for Conducting and Reporting Mixed Research in the Field of Counseling and Beyond. Journal of Counseling & Development. 2010;88:61-9.
10. Moher D, Hopewell S, Schulz KF, et al. CONSORT 2010 Explanation and Elaboration: Updated guidelines for reporting parallel group randomised trials. J Clin Epidemiol. 2010;63:e1-37.
11. Montgomery P, Grant S, Mayo-Wilson E, et al. Reporting randomised trials of social and psychological interventions: the CONSORT-SPI 2018 Extension. Trials. 2018;19:407.
12. Prady SL, Richmond SJ, Morton VM, et al. A systematic evaluation of the impact of STRICTA and CONSORT recommendations on quality of reporting for acupuncture trials. PLoS One. 2008;3:e1577.
13. Ros T, Enriquez-Geppert S, Zotev V, et al. Consensus on the reporting and experimental design of clinical and cognitive-behavioural neurofeedback studies (CRED-nf checklist). Brain. 2020;143:1674-85
14. Tate RL, Perdices M, Rosenkoetter U, et al. The Single-Case Reporting Guideline In BeEhavioural Interventions (SCRIBE) 2016 Statement. J Clin Epidemiol. 2016;73:142-52
15. Virués-Ortega J, Moreno-Rodríguez R. Guidelines for clinical case reports in behavioral clinical Psychology. International journal of clinical and health psychology. 2008;8:765-77.
16. Witkiewitz K, Finney JW, Harris AH, et al. Guidelines for the reporting of treatment tfor alcohol use disorders. Alcohol Clin Exp Res. 2015;39:1571-81.

***b. Quality/risk of bias tools (n=24)***

1. Assendelft WJ, Hay EM, Adshead R, et al. Corticosteroid injections for lateral epicondylitis: a systematic overview. Br J Gen Pract. 1996;46:209-16.
2. Chalmers TC, Smith H Jr, Blackburn B, et al. A method for assessing the quality of a randomized control trial. Control Clin Trials 1981;2:31-49.
3. Chambless DL, Hollon SD. Defining empirically supported therapies. J Consult Clin Psychol. 1998;66(1):7-18.
4. Cuijpers P, van Straten A, Bohlmeijer E, et al. The effects of psychotherapy for adult depression are overestimated: a meta-analysis of study quality and effect size. Psychol Med. 2010;40:211-23.
5. Cho MK, Bero LA. Instruments for assessing the quality of drug studies published in the medical literature. JAMA 1994;272:101-104.
6. D'Andrea E, Vinals L, Patorno E, et al. How well can we assess the validity of non-randomised studies of medications? A systematic review of assessment tools. BMJ Open. 2021;11:e043961.
7. Downs SH, Black N. The feasibility of creating a checklist for the assessment of the methodological quality both of randomised and non-randomised studies of health care interventions. J Epidemiol Community Health 1998;52:377–84.
8. Higgins JP, Altman DG, Gotzsche PC, et al. The Cochrane Collaboration's tool for assessing risk of bias in randomised trials. BMJ. 2011;343:d5928.
9. Jadad AR, Moore RA, Carroll D, et al. Assessing the quality of reports of randomized clinical trials: is blinding necessary? Control Clin Trials 1996;17:1-12.
10. Kmet L M, Lee R C, Cook L S. Standard quality assessment criteria for evaluating primary research papers from a variety of fields. Edmonton: Alberta Heritage Foundation for Medical Research (AHFMR). AHFMR - HTA Initiative #13. 2004. Available at: <https://www.ihe.ca/advanced-search/standard-quality-assessment-criteria-for-evaluating-primary-research-papers-from-a-variety-of-fields>
11. Kocsis JH, Gerberb AJ, Milroda B, et al. A new scale for assessing the quality of randomized clinical trials of psychotherapy. Comprehensive Psychiatry 2009; 51(3): 319–24.
12. Moher D, Jadad AR, Nichol G, et al. Assessing the quality of randomised controlled trials: an annotated bibliography of scales and checklists. Controlled Clinical Trials 1995; 16: 62–73.
13. Moncrieff J, Churchill R, Drummond C, et al. Development of a quality assessment instrument for trials of treatments for depression and neurosis. International Journal of Methods in Psychiatric Research 2001;10(3):126‐133.
14. National Heart, Lung, and Blood Institute (NIH). Study quality assessment tools, quality assessment of controlled intervention studies. Available at: <https://www.nhlbi.nih.gov/health-topics/study-quality-assessment-tools>
15. Ost LG. Efficacy of the third wave of behavioral therapies: a systematic review and meta-analysis. Behav Res Ther. 2008;46:296-321.
16. Petrak F, Hardt J, Nickel R, et al. Checkliste zur Bewertung der wissenschaftlichen Qualität kontrollierter psychotherapeutischer Interventionsstudi- en (CPI). Psychotherapeut 1999; 44: 390-3.
17. Pluye P, Robert E, Cargo M, et al, 2011. Proposal: A mixed methods appraisal tool for systematic mixed studies reviews. Available at: <http://mixedmethodsappraisaltoolpublic.pbworks.com>
18. Scottish Intercollegiate Guidelines Network (2011) Methodology Checklist 2: Randomised controlled trials. Scottish Intercollegiate Guidelines Network. Available at: <https://bmjopen.bmj.com/content/suppl/2015/10/01/bmjopen-2015-008807.DC1/bmjopen-2015-008807supp2.pdf>
19. Sindhu F, Carpenter L, Seers K. Development of a tool to rate the quality assessment of randomized controlled trials using a Delphi technique. Journal of Advanced Nursing 1997; 25: 1262-8.
20. Sterne JA, Hernan MA, Reeves BC, et al. ROBINS-I: a tool for assessing risk of bias in non-randomised studies of interventions. BMJ. 2016;355:i4919.
21. Sterne JAC, Savovic J, Page MJ, et al. RoB 2: a revised tool for assessing risk of bias in randomised trials. BMJ. 2019;366:l4898.
22. Stone JC, Glass K, Clark J, et al. The MethodologicAl STandards for Epidemiological Research (MASTER) scale demonstrated a unified framework for bias assessment. J Clin Epidemiol. 2021;134:52-64
23. Tarrier N, Wykes T. Is there evidence that cognitive behaviour therapy is an effective treatment for schizophrenia? A cautious or cautionary tale? Behav Res Ther. 2004;42:1377-401.
24. Thomas H, Ciliska D, Micucci S, et al. Effectiveness of physical activity enhancement and obesity prevention programs in children and youth (Report) Hamilton, ON: Effective Public Health Practice Project (EPHPP); 2004 [cited 2021 June 21]. Available from: <http://old.hamilton.ca/phcs/ephpp/Research/Summary/2004/HealthyWeightsFull2004.pdf>.

***c. Systematic review handbooks (n=2)***

1. Higgins JPT, Green S (editors). Cochrane Handbook for Systematic Reviews of Interventions Version 5.0.0 [updated February 2008]. The Cochrane Collaboration, 2008. Available from [www.cochrane-handbook.org](http://www.cochrane-handbook.org).
2. Higgins JPT, Thomas J, et al (editors). Cochrane Handbook for Systematic Reviews of Interventions version 6.2 (updated February 2021). Cochrane, 2021. Available from [www.training.cochrane.org/handbook](http://www.training.cochrane.org/handbook).

***d. Methodological guidance on intervention integrity (n=14)***

1. Bellg AJ, Borrelli B, Resnick B, et al. Enhancing treatment fidelity in health behavior change studies: best practices and recommendations from the NIH Behavior Change Consortium. Health Psychol. 2004;23:443-51.
2. Borrelli B, Sepinwall D, Ernst D, et al. A new tool to assess treatment fidelity and evaluation of treatment fidelity across 10 years of health behavior research. J Consult Clin Psychol. 2005;73:852-60.
3. Capin P, Walker MA, Vaughn S, et al. Examining how treatment fidelity is supported, measured, and reported in K–3 reading intervention research. Educational Psychology Review. 2018;30:885-919.
4. Dumas JE, Lynch AM, Laughlin JE, et al. Promoting intervention fidelity. Conceptual issues, methods, and preliminary results from the EARLY ALLIANCE prevention trial. Am J Prev Med. 2001;20:38-47.
5. Glasziou P, Meats E, Heneghan C, et al. What is missing from descriptions of treatment in trials and reviews? BMJ. 2008;336:1472-4.
6. Kazdin AE. Comparative outcome studies of psychotherapy: methodological issues and strategies. J Consult Clin Psychol. 1986;54:95-105.
7. Leeuw M, Goossens ME, de Vet HC, et al. The fidelity of treatment delivery can be assessed in treatment outcome studies: a successful illustration from behavioral medicine. J Clin Epidemiol. 2009;62:81-90
8. Mayo-Wilson E. Reporting implementation in randomized trials: proposed additions to the consolidated standards of reporting trials statement. Am J Public Health. 2007;97:630-3.
9. McCambridge J, Witton J, Elbourne DR. Systematic review of the Hawthorne effect: New concepts are needed to study research participation effects. Journal of Clinical Epidemiology. 2014;67:267-77.
10. Miller S, Binder J. The effects of manual-based training on treatment fidelity and outcome: A review of the literature on adult individual psychotherapy. Psychotherapy: Theory, Research, Practice, Training. 2002;39:184-98.
11. Perepletchikova F, Kazdin AE. Treatment integrity and therapeutic change: issues and research recommendations. Clin Psychol Sci Pract. 2005;12:365-83.
12. Robins JL, Jallo N, Kinser PA. Treatment Fidelity in Mind-Body Interventions. J Holist Nurs. 2019;37:189-99.
13. Robins JL, Jallo N, Kinser PA. Treatment fidelity in mind-body interventions. J Holist Nurs. 2019;37:189-99.
14. Whitlock EP, Orleans CT, Pender N, et al. Evaluating primary care behavioral counseling interventions: an evidence-based approach. Am J Prev Med. 2002;22:267-84.
