# Supplementary material for: The RIPI-f (Reporting Integrity of Psychological Interventions delivered face-to-face) checklist was developed to guide reporting of treatment integrity in face-to-face psychological interventions
Source: J Clin Epidemiol. Author manuscript; Available in PMC 2024 Jun 21. (PMC11192047; doi:10.1016/j.jclinepi.2022.07.013)
Supplement: 3 [file NIHMS2000500-supplement-3.pdf]

What is this project about?

1. Our aim

[illegible]

Treatment integrity refers to the degree to which an intervention is delivered as intended.

[illegible]

2. Who coordinates this project?

[illegible]

Project team:

[illegible]

Research declarations

1. Has this project been approved by an ethical committee?

[illegible]

2. What will happen with my personal data collected during the study?

[illegible]

3. Are there any risks or costs associated with being in the study?

[illegible]

4. Can I tell other people about the study?

[illegible]

5. Can I leave the study once I have started?

[illegible]

6. What if I need further information or I have a complaint about the study?

[illegible]

What is my contribution?

1. Who can take part in the study

[illegible]

2. Your contribution

[illegible]

Round 1 of the survey

[illegible]

Round 2 of the survey

[illegible]

Consensus meeting

[illegible]

Consent to participate in the survey

I confirm that I understand the information provided for this study and I agree to take part.

- Yes
- No

[illegible]

[illegible]

[illegible]

- Yes
- No

[illegible]

Personal information

[illegible]

1. Year of birth

2. What is your current gender identity?

- Woman
- Man
- Non-binary
- Genderqueer
- A gender identity not listed here (please specify)
- Prefer not to state

3. Category that best describes your current role:

- Health professional: psychologist
- Health professional: physician
- Health professional: other. Detail:
- Researcher: conduct of primary studies of psychological interventions
- Researcher: systematic reviews or epidemiological studies
- Other (such as journalist/editor)

4. In which country do you currently reside?

5. What is your academic background?

- Psychology
- Medicine
- Other (please detail):

6. What is your highest academic degree?

- University degree (Bachelor, Master)
- PhD or doctoral degree
- Professor or equivalent

7. How long have you held a clinical role in the **last ten years**?

- Less than 2 years
- 2-4 years
- 5-10 years
- 10 years
- Not applicable / I am not a clinician

8. How long have you held a research role **in the last ten years**?

- Less than 2 years
- 2-4 years
- 5-9 years
- 10 years
- Not applicable / I am not a researcher

9. Please provide your number of peer reviewed publications **in the last five years**:

- None
- 1-5 publications
- 6-10 publications
- 11-20 publications
- More than 20 publications
- Not applicable / I am not a researcher

How to complete the survey

[illegible]

[illegible]

1. Justification of why the intervention can work (WHY)

[illegible]

The authors provide a clear description of...

	1	2	3	4	5	6	7	8	9	Low	Mod	High
1. ... the rationale of why the intervention is expected to work.												
2. ... the theory, models, or hypothesis by which the intervention is expected to work.												
3. ... the specific mechanisms by which the intervention is expected to work.												
4. ... the goals or aims of the key elements of the intervention.												

Please provide comments or add other aspects that may be important to assess treatment integrity with regards to the justification of the intervention:

2. What was done during the intervention (WHAT)

[illegible]

The authors provide a clear description of...

	1	2	3	4	5	6	7	8	9	Low	Mod	High
1. ... the different components of the intervention. [illegible]												
2. ... the materials, that is, physical objects, informative materials or digital media used [illegible]												
3. ... the processes used to deliver the main intervention, including any enabling or support activities.												

Please provide comments or add other aspects that may be important to assess treatment integrity with regards to the content of the intervention:

2.1. Specific aspects related to the materials used during the intervention

[illegible]

The authors provide a clear description of...

	1	2	3	4	5	6	7	8	9	Low	Mod	High
1. ... the materials used by the intervention provider during the intervention.												
2. ... the materials used by the participants during the intervention [illegible]												
3. ... the materials used for the training of intervention providers.												
4. ... the type of materials used during the intervention [illegible]												
5. ... when the materials were used [illegible]												
6. ... where the materials can be accessed [illegible]												
7. ... the protocol or manual for the delivery of the intervention.												

Please provide comments or add other aspects that may be important to assess treatment integrity with regards to the specific materials used during the intervention:

3. Person that provided the interventions (WHO)

[illegible]

The authors provide a clear description of...

	1	2	3	4	5	6	7	8	9	Low	Mod	High
1. ... the number of providers that delivered the intervention.												
2. ... how the competence of the providers with the intervention was assessed.												
3. ... the competence of the providers with the intervention.												
4. ... the providers allegiance to the intervention [illegible]												

Please provide comments or add other aspects that may be important to assess treatment integrity with regards to the treatment provider:

4. Mode of delivery of the interventions (HOW)

[illegible]

The authors provide a clear description of...

	1	2	3	4	5	6	7	8	9	Low	Mod	High
1. ... the mode of delivery [illegible]												
2. ... the number of therapists per patient (one or more than one).												
3. ... the number of participants per therapist (individual therapy or group therapy).												

Please provide comments or add other aspects that may be important to assess treatment integrity with regards to the mode of delivery:

5. Locations where the interventions were delivered (WHERE)

[illegible]

The authors provide a clear description of...

	1	2	3	4	5	6	7	8	9	Low	Mod	High
1. ... the number of sites where the interventions were delivered.												
2. ... the type of setting in which the interventions were delivered [illegible]												
3. ... the infrastructure that was required to deliver the interventions [illegible]												

Please provide comments or add other aspects that may be important to assess treatment integrity with regards to the location of the intervention:

6. Planned intensity of the interventions (WHEN and HOW MUCH)

[illegible]

The authors provide a clear description of...

	1	2	3	4	5	6	7	8	9	Low	Mod	High
1. ... the duration (for example, in minutes) of each session.												
2. ... the number of sessions.												
3. ... the schedule of the sessions (time between sessions).												
4. ... the total duration of the intervention period [illegible]												

Please provide comments or add other aspects that may be important to assess treatment integrity with regards to the planned intensity of the interventions:

7. TAILORING of the intervention

[illegible]

The authors provide a clear description of the following aspects:

	1	2	3	4	5	6	7	8	9	Low	Mod	High
1. If the intervention was tailored to the patient.												
2. The rationale for tailoring the intervention.												
3. What was tailored.												
4. How the intervention was tailored.												

Please provide comments or add other aspects that may be important to assess treatment integrity with regards to the tailoring of the intervention:

8. Allowed modifications of the intervention

[illegible]

The authors provide a clear description of...

	1	2	3	4	5	6	7	8	9	Low	Mod	High
1. ... the changes in the intervention that were allowed.												
2. ... the reasons for admitting modifications in the intervention.												

Please provide comments or add other aspects that may be important to assess treatment integrity with regards to the modifications allowed in the intervention:

9. Attempts to maintain or increase the intervention fidelity

[illegible]

The authors provide a clear description of...

	1	2	3	4	5	6	7	8	9	Low	Mod	High
1. ... the methods that were used to assess the providers' fidelity with the intervention [illegible]												
2. ... the efforts that were implemented to maintain or increase the providers' fidelity with the intervention.												

Please provide comments or add other aspects that may be important to assess treatment integrity with regards to the measures to maintain or increase the intervention fidelity:

10. Measures to maintain or increase the participants' adherence

[illegible]

The authors provide a clear description of...

	1	2	3	4	5	6	7	8	9	Low	Mod	High
1. ... the methods that were used to assess the participants' adherence.												
2. ... the efforts that were implemented to maintain or increase the participants' adherence.												

Please provide comments or add other aspects that may be important to assess treatment integrity with regards to the measures taken to increase the participants' adherence:

11. Observed fidelity of the providers with the interventions

[illegible]

The authors provide a clear description of...

	1	2	3	4	5	6	7	8	9	Low	Mod	High
1. ... the observed fidelity with the interventions expressed quantitatively [illegible]												
2. ... a judgement if the observed fidelity was low enough to distort the study results.												
3. ... the full list of deviations from the intended interventions.												
4. ... the consistency of the delivery of the intervention across providers.												
5. ... the consistency of the delivery of the intervention across sites.												
6. ... the reasons that may explain an observed low fidelity in providers.												

Please provide comments or add other aspects that may be important to assess treatment integrity with regards to the observed fidelity of the providers:

11.1. Providers' deviations from the intended interventions

[illegible]

The authors provide a clear description of...

	1	2	3	4	5	6	7	8	9	Low	Mod	High
1. ... the number of providers with imperfect compliance with the interventions.												
2. ... the observed duration (for example, in minutes) of each session.												
3. ... the observed number of sessions.												
4. ... the observed schedule of the sessions (time between sessions).												
5. ... the observed total duration of the intervention period.												
6. ... the number of providers that stopped delivering the intended interventions [illegible]												
7. ... the number of providers that stopped delivering the intended interventions and switched to other active interventions.												
8. ... the number of providers that stopped delivering the intended interventions and switched to the comparator intervention.												
9. ... the number of providers that added non-planned interventions [illegible]												
10. ... the deviations from the intended intervention [illegible]												

11.2. Description of specific reasons that may explain an observed low fidelity of providers

Following you will find specific **reasons that might be relevant to explain the low fidelity of the providers**. Please rate their importance to assess treatment integrity and your confidence in your rating.

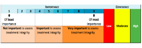

**The authors provide a clear description of the following aspects:**	1	2	3	4	5	6	7	8	9	Low	Mod	High
1. Similarity of study protocols across sites.												
2. Providers differences in their preference for the specific intervention (allegiance).												
3. Numbers of participants with adverse events.												

Please provide comments or add other aspects that may be important to assess treatment integrity with regards to the reasons indicated that may explain low fidelity in the providers:

12. Observed adherence of the participants to the interventions

Following you will find aspects that might be relevant to assess **the observed adherence of the participants to the interventions**. Please rate their importance to assess treatment integrity and your confidence in your rating.

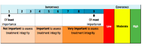

**The authors provide a clear description of…**	1	2	3	4	5	6	7	8	9	Low	Mod	High
1. … the observed adherence to the interventions expressed quantitatively *(for example, the proportion of participants that adhered to the assigned intervention regimen, or the proportion of sessions or hours that the participants missed)*.												
2. … a judgment if the observed adherence was low enough to distort the study results.												
3. … the full list of deviations from the intended interventions.												
4. … the consistency of the participants' adherence to the interventions across providers.												
5. … the consistency of the participants' adherence to the interventions across sites.												
6. … the reasons that may explain an observed low adherence in participants.												

Please provide comments or add other aspects that may be important to assess treatment integrity with regards to the observed adherence of the participants:

12.1.a Participants' deviations from the intended interventions

Following you will find **deviations from the planned interventions in participants** that might be relevant to assess **the observed adherence to the intervention**. The participant is the person receiving the intervention, for example a patient. Please rate their importance to assess treatment integrity and your confidence in your rating.

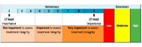

**The authors provide a clear description of…**	1	2	3	4	5	6	7	8	9	Low	Mod	High
1. … the number of participants with or without full adherence.												
2. … the observed duration *(for example, in minutes)* of each session.												
3. … the observed number of attended sessions.												
4. … the observed schedule of the attended sessions *(time between sessions)*.												
5. … the observed total duration of the intervention period.												
6. … … the number of participants that stopped the intervention *(cessation of the intervention)* without adding other interventions but that continued in study.												

Please provide comments or add other aspects that may be important to assess treatment integrity with regards to the participants' deviations from the intended intervention:

12.1.b Participants' deviations from the intended interventions

Following you will find **deviations from the planned interventions in participants** that might be relevant to assess **the observed adherence to the intervention**. The participant is the person receiving the intervention, for example a patient. Please rate their importance to assess treatment integrity and your confidence in your rating.

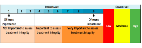

**The authors provide a clear description of…**	1	2	3	4	5	6	7	8	9	Low	Mod	High
7. … the number of participants that withdraw from the study.												
8. … the number of participants that were lost from the study.												
9. … the number of participants that stopped with the intervention *(cessation of the intervention)* and switched to other active interventions.												
10. … the number of participants that stopped with the intervention *(cessation of the intervention)* and switched to the comparator intervention *(crossover)*.												
11. … the number of participants that also received other active intervention not admitted in the study.												
12. … the number of participants that also received the comparator intervention *(contamination)*.												
13. … the number of deviations from the intended intervention that were expected in usual practice or that arose because of the trial context.												

Please provide comments or add other aspects that may be important to assess treatment integrity with regards to the participants' deviations from the intended intervention:

12.2. Description of specific reasons that may explain an observed low adherence in participants

Following you will find specific **reasons that might be relevant to explain the low adherence in participants**. Please rate their importance to assess treatment integrity and your confidence in your rating.

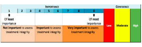

**The authors provide a clear description of the following aspects:**	1	2	3	4	5	6	7	8	9	Low	Mod	High
1. The study was carried out in different sites with different protocols.												
2. Providers with different treatment allegiance, so some of them were not eager to deliver the allocated intervention.												
3. Allocation to an intervention for which the participants had low expectancy for a benefit *(such as a placebo group)*.												
4. Withdrawals from the study due to the presence of adverse events.												

Please provide comments or add other aspects concerning the reasons that may explain a low adherence in participants:

13. Co-interventions

Co-interventions are **any additional diagnostic or therapeutic procedures** to people receiving a particular programme of treatment. Following you will find aspects related with the co-interventions that might be relevant to assess treatment integrity. Please rate their importance to assess treatment integrity and your confidence in your rating.

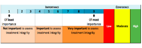

**The authors provide a clear description of…**	1	2	3	4	5	6	7	8	9	Low	Mod	High
1. … the co-interventions that were admitted.												
2. … the co-interventions that were not admitted.												
3. … the admitted co-interventions that were received.												
4. … the not admitted co-interventions that were received.												

Please provide comments or add other aspects that may be important to assess treatment integrity with regards to co-interventions:

Further aspects of treatment integrity

Are there further relevant aspects to assess the integrity of face-to-face psychological interventions? Please feel free to add.

General feedback

If you want to give us a general feedback about our project, you can leave your comments below:

Thank you for completing this survey!

We would like to thank you very much for helping us.

Your answers were transmitted, you may close the browser window or tab now.
