# Supplementary material for: The RIPI-f (Reporting Integrity of Psychological Interventions delivered face-to-face) checklist was developed to guide reporting of treatment integrity in face-to-face psychological interventions
Source: J Clin Epidemiol. Author manuscript; Available in PMC 2024 Jun 21. (PMC11192047; doi:10.1016/j.jclinepi.2022.07.013)
Supplement: 4 [file NIHMS2000500-supplement-4.docx]

# **Appendix 4. Changes to TIDieR**

RIPI-f integrates and enhances the TIDieR checklist [1]. This appendix describes the main changes to TIDieR.

**1. RIPI-f: more complete and specific than TIDieR for face-to-face psychological interventions**

RIPI-f is more exhaustive than TIDieR, allowing for a detailed examination of all the relevant actors and processes for the integrity of psychological interventions [2, 3]. Moreover, RIPI-f proposes specific items instead of general guidance to foster operative, transparent, explicit, and tailored guidance of integrity reporting. This exhaustiveness explains why RIPI-f includes 50 aspects, divided into 12 domains and 16 subdomains.

**2. RIPI-f integrates relevant methodological guidance on psychological intervention integrity**

RIPI-f is the first guideline for reporting the integrity of face-to-face psychological interventions. It broadens the concept of intervention integrity presented in TIDieR and CONSORT-SPI by including psychological and behavioural research [2, 4-6]. Noteworthy is the incorporation of MATD (*Method of Assessing Treatment Delivery*) [7] and Borrelli’s frameworks [2]. The latter is based on the National Institutes of Health (NIH) recommendations for enhancing treatment fidelity in health behaviour change studies [5]. RIPI-f considers many aspects that are omitted in TIDieR but that are central according to recent investigations of intervention integrity [4]. An example is the consideration of the allegiance to the intervention.

**3. Differentiation between participants’ and providers’ contributions to intervention integrity**

TIDieR focuses on the providers’ contribution to integrity, particularly their competence and adherence. However, this partially characterizes psychological interventions’ integrity [7]. On the other hand, RIPI-f also captures the contribution of participants. Moreover, our tool integrates concepts usually confounded by researchers, such as allegiance, motivation, intervention differentiation, receipt, and enactment and expectations [2, 3, 5, 7-11] (see definitions in Box 1: Glossary of terms).

RIPI-f proposes to report the following components separately:

- **Providers**: competencies, allegiance, motivation, therapeutic alliance, awareness of being observed, supervision, adherence, and intervention differentiation. For example, we request the reporting of the providers’ motivation with the client. When a client is difficult to manage or has characteristics that result in early treatment failure, the provider may be more eager to refer this client elsewhere, which translates into lower integrity [3].
- **Participants**: receipt and enactment of the intervention, expectations, awareness of being observed, supervision, and adherence [2, 3, 5, 7-11]. We include the participants’ expectations for the intervention as this can impact the intervention integrity [3]. The tool also considers attempts to enhance the participants’ knowledge about the intervention, its therapeutic goals, and potential side effects. These efforts may influence the participant’s acceptance of the intervention and, thus, the integrity [3].

Our tool requests a separate reporting of these aspects as any breakdown can compromise integrity [2, 3]. For example, providers may adhere to the intervention protocol and be skilled in the delivery. However, their lack of empathy for the client can compromise the intervention integrity and, thus, its effects. On the other hand, the intervention can be wrongly judged as ineffective when there is a low participants’ receipt because they did not know to implement the competencies learned [2]. Moreover, even if the participants understand how to apply a particular intervention, they may not try the intervention between sessions; this may lead the researcher to conclude that the intervention was not effective, while there was poor integrity [2]. Inattention to intervention receipt by the participants has been pointed out as a relevant threat to the study’s validity [2, 8].

**4. Distinction between planned and observed intervention delivery**

Another critical difference from TIDieR is that RIPI-f requests reporting the planned and observed delivery for all relevant aspects. This separate reporting is vital to identifying deviations from the plan and, thus, compromised integrity.

**5. Consideration of peculiarities of psychological interventions**

A vital improvement concerning TIDieR is that RIPI-f requests the reporting of aspects specific to psychological interventions. An example is the providers’ allegiance to the intervention (item 11). As Borreli *et al*. state, “[…], it is also important to hire those who are not only capable of delivering the intervention but also buy into the theoretical foundation” [2, 7]. Thus, it is vital to know if the providers believe in the psychological intervention they deliver.

RIPI-f also requests reporting other factors related to nonspecific intervention effects, particularly the providers’ motivation and the therapeutic alliance (items 12 and 13) and the participants’ receipt, enactment, and expectations (items 15 to 20). These effects can influence the outcomes [2, 12]. However, they are described in just 6% of the articles [2] and are not considered in TIDieR or CONSORT SPI.

**6. More detailed description of the intervention provider**

RIPI-f proposes a more granular and complete description of the provider (Domain Who). This domain contains subdomains, namely, the number of providers, competencies, allegiance, motivation, therapeutic allegiance, motivation, therapeutic alliance, and awareness of being observed (TIDieR does not consider the latter six). In addition, our competence domain is more comprehensive and integrates critical factors for interventions delivered by human providers.

The tool collects aspects needed to demonstrate that the providers had and maintained the skills for successful intervention delivery. Careful recruitment, thorough training, and ongoing supervision are essential to promoting integrity [13]. Thus, RIPI-f requires reporting the providers’ competence, supervision, and performance at the start of the study, during the study, and among providers. This description allows for determining the competence maintenance over the trial. We think that RIPI-f proposes a structured framework to describe how the providers were trained, whether training was standardized across providers, whether the skill acquisition by providers was measured, and whether these skills were maintained over time. Also, RIPI-f highlights the need to describe the staff competencies, as different competencies may lead to the conclusion that a particular intervention is ineffective when, in fact, it might be effective if delivered by competent staff [13, 14] (items 7 to 10).

**7. Differentiation between the integrity assessment plan and how integrity was finally assessed**

RIPI-f emphasizes the need to describe the assessment plan concerning integrity and how integrity was finally assessed. This description should inform the researchers’ use of validated measurement tools and whether deviations from the plan occurred in the assessment. For example, a study protocol may plan to supervise providers daily in both study arms. However, the authors should state whether the supervision finally occurred in one study arm, as this can affect the internal and external study validity.

Another strength is the explicit consideration of whether the providers and participants were aware of the recording of their sessions, as this can influence the intervention delivery [10, 11, 18, 23, 29] (items 14 and 21).

**8. Reporting of co-interventions**

RIPI-f proposes clear guidance to describe the integrity of the co-interventions. We consider that TIDieR guidance on this matter is vague, as this is the only statement presented: “Control interventions and co-interventions are often particularly poorly described; ‘usual care’ is not a sufficient description.”

**Bibliography**

[1] Hoffmann TC, Glasziou PP, Boutron I, Milne R, Perera R, Moher D, et al. Better reporting of interventions: template for intervention description and replication (TIDieR) checklist and guide. BMJ. 2014;348:g1687.

[2] Borrelli B, Sepinwall D, Ernst D, Bellg AJ, Czajkowski S, Breger R, et al. A new tool to assess treatment fidelity and evaluation of treatment fidelity across 10 years of health behavior research. J Consult Clin Psychol. 2005;73:852-60.

[3] Perepletchikova F, Kazdin AE. Treatment integrity and therapeutic change: issues and research recommendations. Clin Psychol Sci Pract. 2005;12:365-83.

[4] Capin P, Walker MA, Vaughn S, Wanzek J. Examining how treatment fidelity is supported, measured, and reported in K–3 reading intervention research. Educational Psychology Review. 2018;30:885-919.

[5] Bellg AJ, Borrelli B, Resnick B, Hecht J, Minicucci DS, Ory M, et al. Enhancing treatment fidelity in health behavior change studies: best practices and recommendations from the NIH Behavior Change Consortium. Health Psychol. 2004;23:443-51.

[6] Miller WR, Rollnick S. The effectiveness and ineffectiveness of complex behavioral interventions: impact of treatment fidelity. Contemp Clin Trials. 2014;37:234-41.

[7] Leeuw M, Goossens ME, de Vet HC, Vlaeyen JW. The fidelity of treatment delivery can be assessed in treatment outcome studies: a successful illustration from behavioral medicine. J Clin Epidemiol. 2009;62:81-90.

[8] Kazdin AE. Comparative outcome studies of psychotherapy: methodological issues and strategies. J Consult Clin Psychol. 1986;54:95-105.

[9] Moncher FJ PR. Treatment fidelity in outcome studies. Clin Psychol Rev. 1991;11:247e66.

[10] Yeaton WH, Sechrest L. Critical dimensions in the choice and maintenance of successful treatments: strength, integrity, and effectiveness. J Consult Clin Psychol. 1981;49:156-67.

[11] Lichstein KL, Riedel BW, Grieve R. Fair tests of clinical trials: a treatment implementation model. Adv Behav Res Ther. 1994;16:1-29.

[12] Klein DN, Schwartz JE, Santiago NJ, Vivian D, Vocisano C, Castonguay LG, et al. Therapeutic alliance in depression treatment: controlling for prior change and patient characteristics. J Consult Clin Psychol. 2003;71:997-1006.

[13] Dumas JE, Lynch AM, Laughlin JE, Phillips Smith E, Prinz RJ. Promoting intervention fidelity. Conceptual issues, methods, and preliminary results from the EARLY ALLIANCE prevention trial. Am J Prev Med. 2001;20:38-47.

[14] Waltz J, Addis ME, Koerner K, Jacobson NS. Testing the integrity of a psychotherapy protocol: assessment of adherence and competence. J Consult Clin Psychol. 1993;61:623-30.
