# Supplementary material for: The RIPI-f (Reporting Integrity of Psychological Interventions delivered face-to-face) checklist was developed to guide reporting of treatment integrity in face-to-face psychological interventions
Source: J Clin Epidemiol. Author manuscript; Available in PMC 2024 Jun 21. (PMC11192047; doi:10.1016/j.jclinepi.2022.07.013)
Supplement: 5 [file NIHMS2000500-supplement-5.docx]

# Appendix 5. Terminology applied to a behaviour change intervention trial

| **Trial aim:** to determine the effectiveness of a behaviour change intervention targeting diet for preventing migraine in adults.  **Intervention content:** behaviour change intervention that includes teaching participants to prepare recipes avoiding products that increase the risk of migraine.  **Adherence of providers:** degree to which the providers followed the teaching manual and the planned procedures during the actual delivery of the intervention.  **Receipt of the intervention by the participants:** the recipes are discussed with the participants during the intervention to confirm that they understood how to prepare the meals.  **Enactment of the intervention by the participants:** it was checked daily that the study participants had been able to prepare the meals in their real-life settings.  **Adherence of participants:** the extent to which the study participants performed the tasks definitive of the intervention, that is, whether they ate the prepared meals.  **Intervention efficacy:** the extent to which the intervention influences the clinical endpoint of interest (frequency of episodes of migraine).  This trial highlights two messages. First, both the providers' and participants’ behaviours contribute to intervention integrity[1]. Second, providers’ adherence, participants’ adherence, receipt, and enactment should be reported separately. The participants’ enactment of the intervention is often confounded with their adherence to the intervention or with the intervention efficacy. However, the enactment can be adequate (the participants correctly prepare the meals) with insufficient participant adherence (they ultimately do not eat the “well-prepared” meals) or poor intervention efficacy (participants correctly prepare and eat the meals, but migraines are not prevented). The separate reporting can explain the lack of efficacy. |
| --- |

**Bibliography**

[1] Dumas JE, Lynch AM, Laughlin JE, Phillips Smith E, Prinz RJ. Promoting intervention fidelity. Conceptual issues, methods, and preliminary results from the EARLY ALLIANCE prevention trial. Am J Prev Med. 2001;20:38-47.
